# A Review of Electrolyte Additives in Vanadium Redox Flow Batteries

**DOI:** 10.3390/ma16134582

**Published:** 2023-06-25

**Authors:** Wenxin Tian, Hao Du, Jianzhang Wang, Jan J. Weigand, Jian Qi, Shaona Wang, Lanjie Li

**Affiliations:** 1 CAS Key Laboratory of Green Process and Engineering, National Engineering Research Center of Green Recycling for Strategic Metal Resources, Institute of Process Engineering, Chinese Academy of Sciences, Beijing 100190, China; tianwenxin21@ipe.ac.cn (W.T.); hdu@ipe.ac.cn (H.D.); 2 University of Chinese Academy of Sciences, Beijing 100049, China; 3 Faculty of Chemistry and Food Chemistry, TU Dresden, 01062 Dresden, Germany; jianzhang.wang@mailbox.tu-dresden.de (J.W.); jan.weigand@tu-dresden.de (J.J.W.); 4 Chengde Vanadium Titanium New Material Co., Ltd., Chengde 067100, China; qj0011@126.com

**Keywords:** vanadium redox flow batteries, electrolyte additives, electrochemical performance, complexation, electrostatic repulsion, growth inhibition, modifying electrode

## Abstract

Vanadium redox flow batteries (VRFBs) are promising candidates for large-scale energy storage, and the electrolyte plays a critical role in chemical–electrical energy conversion. However, the operating temperature of VRFBs is limited to 10–40 °C because of the stability of the electrolyte. To overcome this, various chemical species are added, but the progress and mechanism have not been summarized and discussed yet. This review summarizes research progress on electrolyte additives that are used for different purposes or systems in the operation of VRFBs, including stabilizing agents (SAs) and electrochemical mass transfer enhancers (EMTEs). Additives in vanadium electrolytes that exhibit microscopic stabilizing mechanisms and electrochemical enhancing mechanisms, including complexation, electrostatic repulsion, growth inhibition, and modifying electrodes, are also discussed, including inorganic, organic, and complex. In the end, the prospects and challenges associated with the side effects of additives in VRFBs are presented, aiming to provide a theoretical and comprehensive reference for researchers to design a higher-performance electrolyte for VRFBs.

## 1. Introduction

The global energy structure is gradually changing from non-renewable energy sources, such as fossil fuels, with high consumption and pollution, to green and low-carbon renewable energy sources. According to the data published by National Development and Reform Commission on 22 September 2022, the installed capacity of renewable energy in China has exceeded 1.1 billion kilowatts and is expected to exceed that of coal power and become the first major power source by 2030. However, renewable energy sources have such problems as discontinuity, instability, high abandoned wind/light rate, and difficulty in frequency/peak regulation of power grids, which may not be able to meet the growing electricity demand. Thus, large-scale and long-time energy storage technologies are urgently needed [1,2,3,4,5]. Among many energy storage technologies, the vanadium redox flow battery (VRFB) has high safety, long cycle life, good charging and discharging performance, rapid response, stable capacity, and low life cycle costs [1,6,7,8,9,10,11], which makes it the largest, most technologically advanced, and closest-to-industrialization liquid flow battery [12,13,14,15,16,17,18]. At present, the world’s largest energy storage project, a 100 MW/400 MWh vanadium battery, has been successfully connected to the grid in Dalian, China and continuous GWh-level projects have submitted bids for construction, indicating great progress in the industrialization of VRFBs.

**Figure 1:**
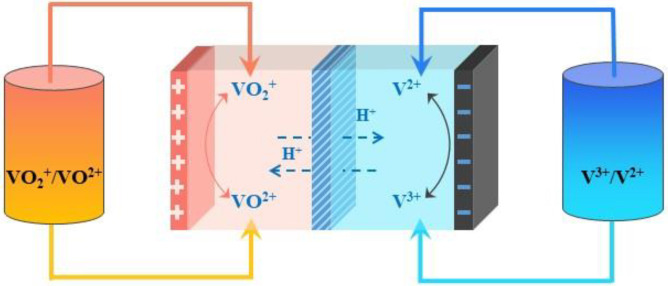
Vanadium redox flow battery schematic.

VRFBs, first proposed by Skyllas-Kazacos in 1986 [19], consist of three key factors: electrodes, an ion exchange membrane, and electrolytes [20,21,22,23]. Among them, the electrolyte, as the core part of the vanadium battery system, greatly affects the energy density and overall performance of the battery. It can generally be divided into positive and negative electrolytes, which correspond to the sulfuric acid solutions of the V(IV)/V(V) and V(II)/V(III) redox couples in Equations (1)–(3) [24], respectively. The same elements at different oxidation states can be converted to one another at the electrodes, achieving the chemical–electrical energy conversion, as shown in Figure 1.

(1) $$\mathrm{Positive}\;\mathrm{electrode}:\;VO^{2+}-e^{-}+H_{2}O⇋VO_{2}^{+}+2H^{+}$$

(2) $$\mathrm{Negative}\;\mathrm{electrode}:\;V^{3+}+e^{-}⇋V^{2+}$$

(3) $$\mathrm{Overall}\;\mathrm{reaction}:\;VO^{2+}+V^{3+}+H_{2}O⇋VO_{2}^{+}+V^{2+}+2H^{+}$$

The vanadium electrolyte is generally prepared through the methods of physical dissolution, chemical reduction, electrolysis, and chemistry–electrolysis coupling [25] Among them, the chemistry–electrolysis coupling is the dominant method, which takes high-purity V_2_O_5_ as the raw material and adds reducing agents such as H_2_C_2_O_4_ [26], SO_2_, [27], and elemental sulfur to the sulfuric acid to prepare the V(IV) electrolyte, and then reduces it by electrolysis to obtain the V(III) electrolyte. If the operating temperature of the vanadium electrolyte is higher than 40 °C or lower than 10 °C, both the electrolyte stability and energy density of vanadium batteries will decrease, accompanied by capacity loss and battery failure [28]. To solve this problem, additives are added to the electrolyte [29] to improve its stability and optimize the electrochemical kinetics, so as to expand the operating temperature range and raise the energy density of the VRFB. For example, the introduction of ammonium dihydrogen phosphate [30] and acidic amino acid [31] as additives can enhance the high-temperature stability of electrolytes, and ammonium and α-lactose monohydrate [32] have been used to improve the low-temperature stability of electrolytes. Additionally, additives such as polyacrylic acid (PAA) [33] can strengthen electrochemical mass transfer.

Additives in vanadium electrolytes, generally classified as inorganics, organics, and compounds, exhibit different microscopic mechanisms, including complexation, electrostatic repulsion, and growth inhibition. Specifically, while complexation improves anti-precipitation properties by changing the distribution of electron clouds, electrostatic repulsion reduces the agglomeration of V(V) ions by enhancing the dispersion effect of V(V) ions from each other. Growth inhibition, on the other hand, lowers the size of settled particles by impeding the growth kinetics of V_2_O_5_. Furthermore, the electrochemical mass transfer enhancers in vanadium electrolytes mainly perform hydrophilic modification to enhance electrochemical kinetics, such as MSA, which can be adsorbed on the electrodes to increase the active sites. This adsorption behavior can promote redox reaction kinetics in the interface and optimize the electrochemical performance of the VRFB. Therefore, the introduction of additives can effectively increase the operating temperature range of the vanadium electrolyte, providing effective technical support for the large-scale application of the VRFB.

The above-mentioned research on the VRFB indicates its excellent application prospects. However, the relatively low energy density and operating temperature range of VRFBs may limit their large-scale industrial applications [1]. To solve this problem, incorporating additives in the electrolyte to modify their characteristics while retaining their bulk properties has received considerable attention in recent years. Nevertheless, few review articles have been published on this topic. Herein, this article reviews the research progress on vanadium electrolyte additives in recent years, focusing on the effects of inorganic, organic, and composite additives on the improved stability and electrochemical performance of the VRFB. Further, the different mechanisms of each additive are also summarized. Finally, we probe into the future directions and perspectives of research on vanadium electrolytes, hoping to provide a theoretical reference for the in-depth optimization of the VRFB performance.

## 2. The Stability of Vanadium Electrolytes

### 2.1. Effect of Temperature Variation on the Stability

Vanadium in different valence states exists in the electrolyte as hydrated ions with the general formula ${\left[V^{Z+}O_{m}{\left(H_{2}O\right)}_{n}\right]}^{\left(Z-2m\right)-}$. Through a deprotonation endothermic reaction performed by nuclear magnetic resonance (NMR) spectroscopy and density functional theory (DFT) (Equation (4)) [34], Vijayakumar et al. [34,35] demonstrated that hydrated V(V) ([VO_2_(H_2_O)_3_]^+^) is likely to produce H_3_VO_4_ at high temperatures. In this study, H_3_VO_4_ undergoes a condensation reaction through the proton exchange of its active hydroxyl group (Equation (5)) [34], forming a V–O–V bond. This behavior leads to the appearance of V_2_O_5_ precipitation at high temperatures (e.g., 50 °C), which triggers flow field perturbation in the cell, resulting in a lower electrolyte concentration and potential energy, as well as capacity decay [28]. The aggravated cross-contamination of electrolytes, electrolytes’ precipitation, and increased polarization resistance will be observed when the temperature is excessive. In contrast, V(II), V(III), and V(IV) form hydrated ions with a similar structure to the hydrated V(V) in the electrolyte: [V(H_2_O)_6_]^2+^, [V(H_2_O)_6_]^3+,^ and [VO(H_2_O)_5_]^2+^, respectively. However, they did not show significant deprotonation [34]. This is because the number of hydroxyl groups produced in the deprotonation reaction is proportional to the valence of vanadium [34]. Therefore, extreme high temperatures (e.g., 50 °C) can lower the stability of vanadium ions of all valance states, and the hydrated ionic structure of low-valence vanadium has higher stability compared to V(V) [36].

(4) $${\left[VO_{2}{\left(H_{2}O\right)}_{3}\right]}^{+}\overset{∆}{\to }H_{3}VO_{4}+H_{3}O$$

(5) $$2H_{3}VO_{4}\to V_{2}O_{5}+3H_{2}O$$

The solubilities of V(II), V(III), and V(IV) species in sulfuric acid rise with ascending temperature. Therefore, their hydrated ions are unstable and tend to precipitate at low temperatures, reducing the capacity utilization of the battery [20]. When the temperature is −10 °C to −20 °C, while both the polarization resistance of the vanadium electrolyte and the activation energy of the reaction increase, the capacity utilization of the device falls [37]. Briefly, low-temperature conditions are also harmful to the stability of electrolytes, resulting in greater demands for the industrialization of VRFBs in an extremely cold environment. In contrast to lower valence states of vanadium, the solubility of V(V) species descends with increasing temperature [20]. According to Vijayakumar et al. [34], the electrolyte shows slow proton exchange rates at low temperatures without a significant deprotonation reaction, which further confirms that the electrolyte is relatively stable at low temperatures. Furthermore, the electrochemical performance also can be affected by a change in temperature. It has been recently shown that the polarization resistance and ohmic resistance of the VRFB are negatively correlated with the temperature within a given operating temperature range. However, in terms of its conductivity, voltage efficiency, and capacity utilization, the opposite trend was found [28].

In short, V(II), V(III), and V(IV) species exhibit poor stability at quite low temperatures (<10 °C), whereas V(V) is sensitive to precipitation at relatively high temperatures (>40 °C). Temperatures between 10 °C and 40 °C are regarded as the ideal operating temperature range for VRFBs due to the temperature instability of the V electrolyte [38,39]. Furthermore, the behaviors of vanadium species in various valences indicate that the stabilization of the hydrated vanadium cation structure is the core mechanism for enhancing stability.

### 2.2. Effect of Stability Improvement on the Electrochemical Performance

The effect of electrolytes on the electrochemical properties is one of the key indicators used for designing and evaluating stabilizing agents while improving the stability of electrolytes. Some inorganic additives, such as the mixture of 2 wt% (NH_4_)_2_SO_4_ and 1 wt% H_3_PO_4_ [40], may cause varying degrees of capacity loss of the VRFB. The adsorption of long-chain organic additives, such as hexadecyl trimethyl ammonium bromide (HTAB), on ions proliferates the number of macromolecular polymers in solution, increasing the viscosity of the electrolyte [41]. This also leads to lower conductivity and mass transfer. Some heterocyclic compounds can cause irreversible capacity loss while improving the stability of electrolytes, such as phytic acid [42]. Part of the strong oxidizing organic additives can result in energy loss of the VRFB because they are involved in its electrochemical reactions [43]. Part of the highly reductive organic additives can be oxidized by V(V) ions in the positive half-cell electrolyte. This process will generate CO_2_, which gives rise to higher overpotential and lower efficiency of the VRFB, such as that brought about by phosphonoacetic acid and ethylenediaminetetraacetic acid [44]. Furthermore, introducing additives in inappropriate dosages also affects the electrochemical properties [45]. Therefore, it is of great significance to enhance the electrochemistry kinetics to improve stability for further applications.

### 2.3. Characterization of Stability

Characterization methods should not be neglected in stability studies, and researchers ought to take full account of the artificiality of the test methods. Apparent stability phenomena have been found to be an interference in the accurate determination of stability changes in vanadium electrolytes. Over the past decade, researchers have become accustomed to characterizing thermal stability by “precipitation time” in studies on VRFB additives. Nevertheless, Nguyen et al. [45] proposed the phenomenon of “apparent stability”, stating that there was an artifact of the test method due to the oxidation of the additive, with a corresponding partial reduction of V(V) to V(IV). This has also been reported by Wang et al. [46], where the “improvement of stability” is essentially at the cost of energy capacity. The original V(V) solution becomes the mixed solution of V(IV) and V(V) with the changes of state-of-charge (SOC) in the electrolyte. This does not achieve a genuine hindrance to the condensation and polymerization of V(V). According to Nguyen et al. [45], the precipitation rate of V_2_O_5_ is dependent on the SOC of the electrolyte (i.e., the relative concentration of V(V) to total vanadium). Thus, researchers optimized the testing method of stability by raising another indicator—“remaining vanadium concentration”—to improve the accuracy of stability characterization. For example, Jin and Ding et al. [44,47] studied the precipitation time and change of the V(V) concentration in the stability experiment to verify the accuracy of the experimental results. Overall, precipitation time and the remaining vanadium concentration are two major indicators of stability, currently. 

## 3. The Function Mechanisms of Additives

The function mechanisms of additives involved in electrolyte performance improvement are still under investigation. Because additives are mainly classified into stabilizing agents, including complexing agents and electrostatic repulsion agents, and growth inhibitors and electrochemical enhancers, the stabilizing mechanisms and enhancement mechanisms will be discussed separately. Whereas the principal stabilizing mechanisms involve complexation, electrostatic repulsion, and growth inhibition, the enhancement mechanism of electrochemical mass transfer mainly involves additives’ hydrophilic modification of the electrode.

### 3.1. Stabilizing Mechanisms

#### 3.1.1. Complexation

**Figure 2:**
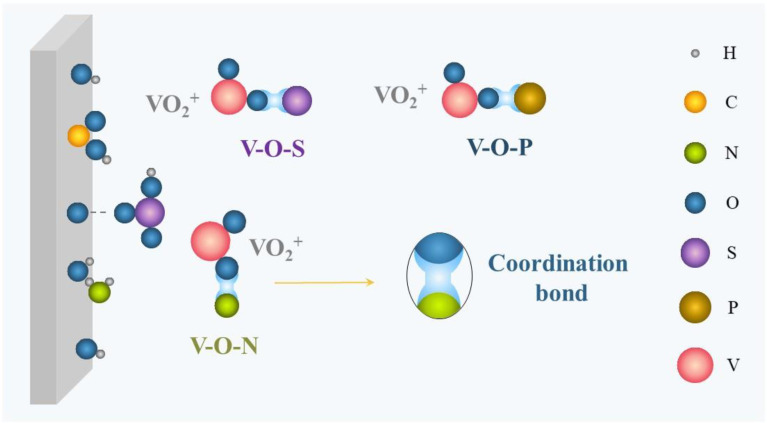
Schematic of complexation.

Complexation is the foremost stabilizing mechanism of additives, and is applied to both inorganic and organic additives. As illustrated in Figure 2, ions (Cl^−^, H_2_PO_4_^−^) or functional groups (-COOH, -NH_2_, -OH, -SO_3_H) carrying lone-pair electrons are capable of coordinating with hydrated vanadium ions in the electrolyte to form a more stable intermediate with V–O–S, V–O–P, V–O–Cl, V–O–N, and so on, thereby effectively reducing the formation of V–O–V and inhibiting V_2_O_5_ precipitation. Subsequently, the electron density of vanadium will increase and the local positive charge of vanadium will decrease [48]. Furthermore, the reaction barrier of forming V–O–X (X is the core element of additives) is generally lower than that of forming V–O–V from V_2_O_5_, which considerably reduces the generation selectivity of V_2_O_5_ precipitation [49,50].

In addition, a large number of studies have shown that complexation behaviors can be impacted by the geometries of hydrated vanadium ions in different valences, the synergistic effect of additives, and other supplementary factors. For one, the geometries of hydrated ions of vanadium in diverse valences are different and the complexing capacity is positively correlated with the stability of the additives to vanadium ions in all valences. Clarifying the complexation of vanadium ions with additives in each valence is necessary for both stabilization maximization and additive selection. For another, adopting synergistic effects can intensify the stabilization effect and maintain a balance between various ions, such as phosphate and ammonium. Finally, other supplementary factors can be involved, including introducing double additives to form a competing relationship, modifying the electrodes of VRFBs, integrating a thermally regenerative electrochemical cycle (TREC) into the VRFBs, and so on. Researchers should be aware of the above-mentioned considerations when selecting additives.

**Figure 3:**
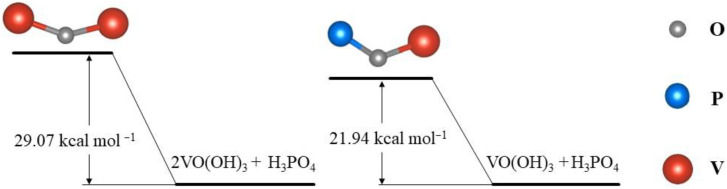
The energy state of the V–O–V bond and V–O–P bond.

Taking H_3_PO_4_ as an example, the transformation path of V(V) in phosphates-added electrolytes is: [VO_2_(H_2_O)_2_]^+^ → [VO(OH)_2_(H_2_O)]^+^ → VO(OH)_3_ [51]. The VO(OH)_3_ intermediate can form a compound containing a V–O–P bond with H_3_PO_4_. Moreover, the activation energy of this reaction is generally lower than that of the formation of the V–O–V bond, which could effectively avoid V_2_O_5_ precipitation, as presented in Figure 3. Furthermore, the dominant form of the anions is H_2_PO_4_^−^ after phosphate additives are added to the positive electrolyte [49]. In this case, due to the partial dimerization of V(V), the sulfate is coordinated to two oxygen atoms in a bridging or bidentate coordination. This can be explained by the coordination of H_3_PO_4_ or by the rearrangement of the complexation pattern due to dimerization.

**Figure 4:**
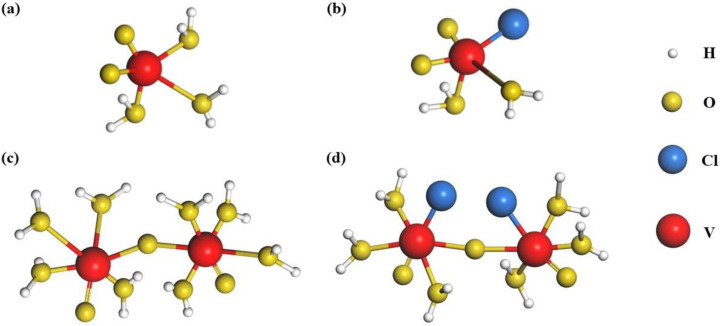
Geometry-optimized structures for [VO_2_(H_2_O)_3_]^+^ compound (**a**), chlorine bonded VO_2_Cl(H_2_O)_2_ compound (**b**), pristine di-nuclear [V_2_O_3_·8H_2_O]^4+^ compound (**c**), and chlorine bonded [V_2_O_3_Cl_2_·6H_2_O]^2+^ compound (**d**).

Taking another case of Cl^−^, it can form the mononuclear complex VO_2_Cl(H_2_O)_2_ with [VO_2_(H_2_O)_3_]^+^ at high temperatures [35]. This process, with low reaction barriers and high priority, will effectively hinder the deprotonation of [VO_2_(H_2_O)_3_]^+^, serving as the first step during the precipitation reaction. Through DFT and NMR spectroscopy analyses, it is further revealed that V_2_O_5_ precipitation also could be formed by the deprotonation of di-nuclear [V_2_O_3_·8H_2_O]^4+^ cations at high temperatures, as seen in Equation (6) [48]. Nevertheless, the chlorine ion can form a stable di-nuclear complex [V_2_O_3_Cl_2_·6H_2_O]^2+^ with [V_2_O_3_·8H_2_O]^4+^ to prevent precipitation [48]. Figure 4 shows the geometry-optimized structures of [VO_2_(H_2_O)_3_]^+^, VO_2_Cl(H_2_O)_2_, [V_2_O_3_·8H_2_O]^4+^, and [V_2_O_3_Cl_2_·6H_2_O]^2+^. The formation of stable structures occurs because Cl_2_ has four groups of lone-pair electrons, which act as electron donors for complexation to the empty orbitals of vanadium ions. Furthermore, the nature of the V–O bond is the attraction of positive and negative charges; thus, the behavior of chlorine complexation makes the V–O bond weaker [48] and the O–H bond stronger, which can lead to a more stable H_2_O molecule and impede the deprotonation of [V_2_O_3_·8H_2_O]^4+^, thereby achieving high stability.

(6) $$2{\left[V_{2}O_{3}\cdot 8H_{2}O\right]}^{4+}\overset{{-8H}^{+}}{\to }{2V}_{2}O_{5}↓+12H_{2}O$$

#### 3.1.2. Electrostatic Repulsion

**Figure 5:**
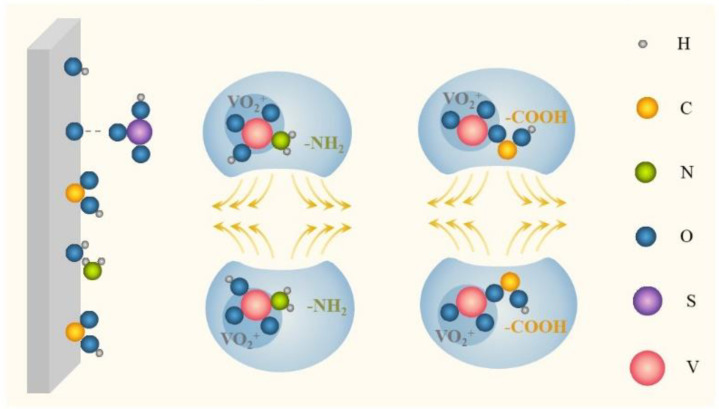
Schematic of electrostatic repulsion.

In electrostatic repulsion, a common stabilizing mechanism, ions or functional groups of additives, can be adsorbed on vanadium ions by electrostatic attraction, mainly including -COOH, -OH, -SO_3_H, -S-, -NH_2_, and so on. This adsorption behavior promotes the formation of ionic agglomerates with vanadium ions as the core element, which can enhance the outer layer charge and generate electrostatic repulsion to varying degrees, as shown in Figure 5. Meanwhile, due to the large core–shell spatial structure, the steric hindrance of this agglomerate encapsulating vanadium ions can strengthen this repulsion effect, making vanadium ions more dispersed, to inhibit precipitation [52]. A proliferation of studies has demonstrated the dominance of electrostatic repulsion in the stabilizing mechanism of organic additives, but the application of this principle in inorganic additives is minimal. More specifically, anionic functional groups produce negatively charged groups (e.g., -COO^−^) in the electrolyte, which brings about electrostatic attraction with the positively charged vanadium ions to form agglomerates. Polar groups (e.g., -NH_2_) tend to be adsorbed on vanadium ions based on the like-dissolves-like theory to generate external charge layers, boosting the repulsion effect [32,33,53].

#### 3.1.3. Growth Inhibition

**Figure 6:**
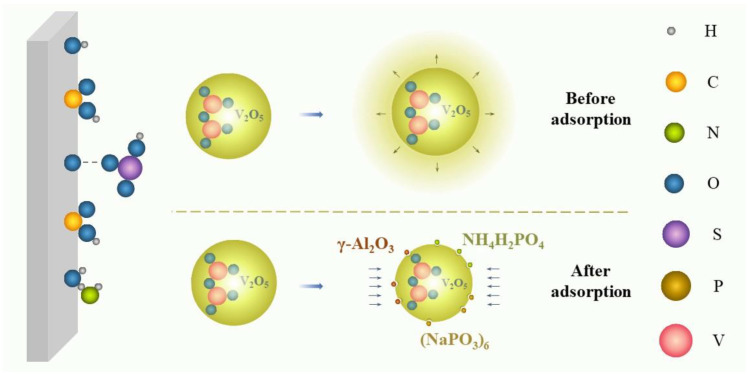
Schematic of growth inhibition.

Growth inhibition, an uncommon stabilizing mechanism, means that some additives can inhibit the growth kinetics of V_2_O_5_ precipitates in the vanadium electrolyte. As shown in Figure 6, the V_2_O_5_ precipitates can generally grow with increasing time without additives. However, after introducing some specific additives, the surfaces of the nucleation sites of the V_2_O_5_ were adsorbed by various molecules to lower the growth rate of V_2_O_5_ precipitates, lengthening the induction time of precipitation and reducing the sizes of V_2_O_5_ particles [43,52,54].

### 3.2. Electrochemical Mass Transfer Enhancement Mechanism

**Figure 7:**
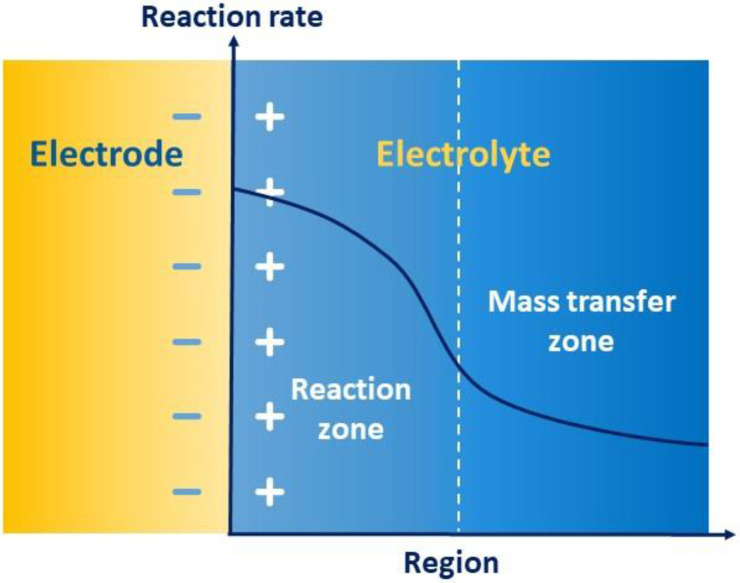
Schematic of the mass transfer of electric double layers.

As Figure 7 shows, the Helmholtz model depicts how the opposite charges can form a set of polar plates in an electrolyte due to mutual attraction in the electrode–electrolyte interface, further forming a capacitor to store energy, which is called the electric double layer. The whole process of redox reaction is divided into a reaction region and a transfer region, whose rates are co-controlled by electron transfer and migrating mass transfer. The high charge gradient in the region of the electric double layer reduces the mass transfer rate of the solid–liquid interface and accelerates the reaction rate. Therefore, in the transfer region, the rate of transfer usually slows down, thus limiting the electrochemical performance, including energy efficiency, capacity retention rate, and the properties of charging and discharging. Accordingly, the mass transfer of electric double layers becomes an important speed-controlled step of electrochemical kinetics.

**Figure 8:**
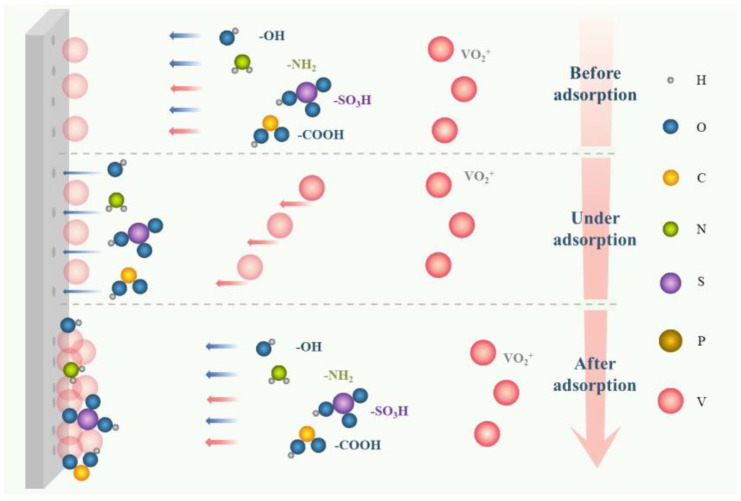
Schematic of the mechanism of electrochemical mass transfer enhancers.

The foremost mechanism of enhancing electrochemical mass transfer is that additives can be adsorbed on the surface of the electrode to promote active sites to form a “hydrophilic modification” to electrodes, mainly including hydroxyl, the sulfonic group, pyridyl, and other hydrophilic functional groups, or certain ions [32,55], as illustrated in Figure 8. This modifying behavior to electrodes can activate the interfacial activity between electrodes and the electrolyte, accelerating both the redox reaction of vanadium ions in all valences and the migration mass transfer. Alternatively, additives, such as taurine, MSA, PPS, benzoyl peroxide, and so on, can reduce the overpotential of the VRFB to facilitate the migrating mass transfer, reducing the resistance of the electric double layer and enhancing the kinetics of redox reactions [56,57,58,59,60].

## 4. Inorganic Additives

**Figure 9:**
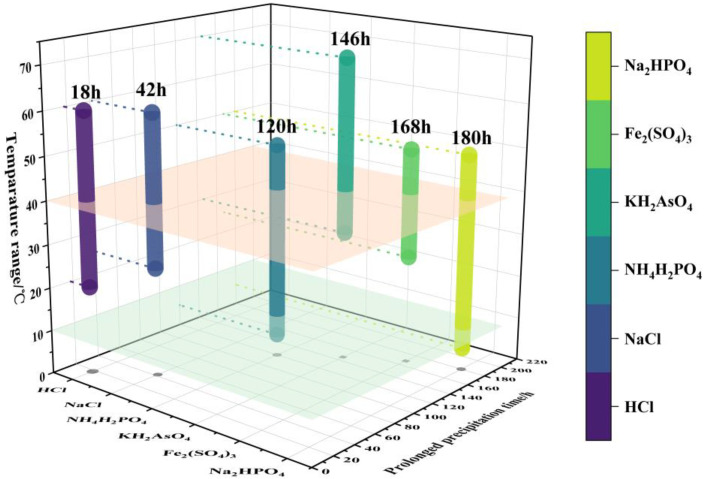
Temperature-window-extended precipitation time distribution of some electrolyte additives.

Table 1 summarizes the inorganic stabilizing agents and electrochemical mass transfer enhancers reported in recent years. As seen in Table 1, inorganic additives of electrolytes mainly include inorganic acids, inorganic salts, and metal oxides, such as HCl, H_3_PO_4_, NaH_2_PO_4_, NaCl, TiO_2_, and γ-Al_2_O_3_. The effects of several typical inorganic additives on prolonging precipitation time and widening temperature windows are illustrated in Figure 9, which indicates that the electrolyte’s precipitation time at a wide temperature range can be extended from 18 to 168 h.

### 4.1. Stabilizing Agents

#### 4.1.1. Complexing Agents

Many studies by the UNSW group [43], the Dalian Institute of Chemical Physics, and other teams [30,54,71,72] have shown that phosphate series additives are one of the major complexing agents. The precipitation time of electrolytes could be extended by phosphate series additives up to six times at high temperatures. Moreover, many commercially available electrolytes are supplemented with small amounts of phosphoric acid [73]. Oldenburg et al. [74] further explored the complexation of vanadium ions of different valences in sulfate–phosphate electrolytes by carrying out geometry optimization calculations of the possible solvation structures of vanadium. Through DFT, it was found that the hydrated V (II) showed a hexa-coordinated octahedron in its geometry and displayed the worst complexing ability. The geometry of hydrated V(III) is similar to that of hydrated V(II), and in addition, because of the difference in free energy between them, the complexing ability of hydrated V(III) is stronger than that of hydrated V(II). The hydrated V(IV), with either octahedral or square-pyramidal geometries, can coordinate with sulfate as well as phosphate, and the complexing ability of phosphate is stronger than that of sulfate. The hydrated V(V) showed a tetrahedral geometry, which has a comparable complexing ability with sulfate and phosphate [74]. Overall, the complexing ability between phosphate series additives and vanadium ions exhibits excellent performance, especially for V(IV) and V(V) ions. In addition to phosphates, chloride additives also have similar stabilizing effects. The behaviors of sulfate–chloride mixed-acid systems have been continuously investigated by Vijayakumar and colleagues from the Pacific Northwest National Laboratory (PNNL) [35,48]. For instance, Chen et al. [69] found that 4% NaCl can stabilize the positive electrolyte for 45 days at 50 °C. Many other complexing agents have also successfully achieved high-temperature stability through complexation, such as Fe_2_(SO_4_)_3_ and KH_2_AsO_4_ [62,63]. Based on the excellent stabilizing effect of H_2_PO_4_^−^ and H_2_AsO_4_^−^, researchers have speculated that other elements of group VA, such as nitrogen, [63] antimony, and bismuth [75], may have similar properties. However, there are few relevant studies, and the specific mechanisms need to be further explored.

**Figure 10:**
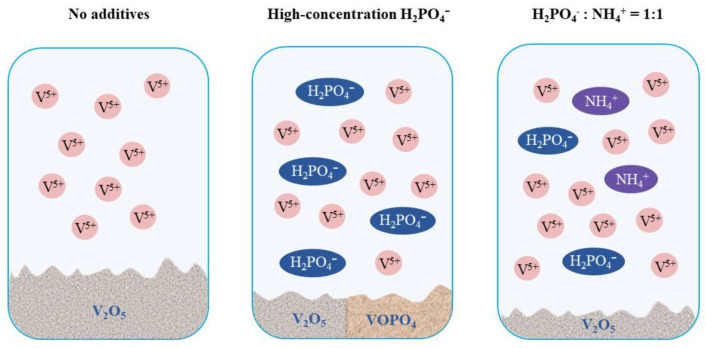
Synergism between H_2_PO_4_^−^ and NH^4+^.

In addition to the introduction of complexing agents alone, there are cases where multiple complexing agents, such as phosphate and ammonium, are introduced to ensure the stabilization effect via synergy and balances. Ding et al. [30] demonstrated that V(V) species strongly coordinated with H_2_PO_4_^−^ so that the formation of V_2_O_5_ precipitates would be theoretically inhibited. However, at higher concentrations of H_3_PO_4_, VOPO_4_ will co-precipitate with V_2_O_5_, which can disrupt the electrolyte stability and limits the maximum concentration of H_3_PO_4_ that may be practically employed [30,54,71]. According to Nguyen et al. [54], this problem could be solved by the synergism between phosphate and ammonium ions. After adding (NH_4_)_3_PO_4_, the precipitate in the electrolyte only contained V_2_O_5_ without the VOPO_4_ phase, as shown in Figure 10. Meanwhile, the best effect can be achieved when the molar ratio of nitrogen to phosphorus is 1:1. Therefore, the rational application of the “synergism” is of great significance for the optimization design of additives to enhance stability.

**Figure 11:**
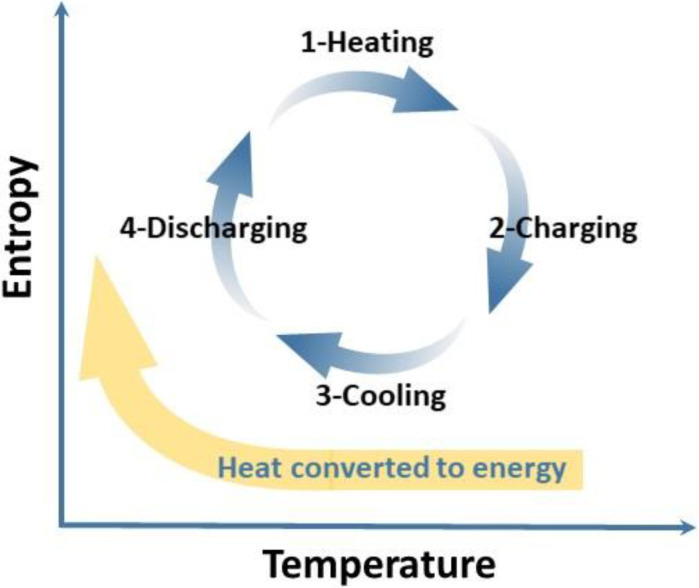
Schematic of the thermodynamic cycle performed by a TREC.

Other supplementary measures have been adopted to maximize the enhancement of stability when introducing complexing agents. For example, MgCl_2_ and NH_4_H_2_PO_4_ were selected as additives to simultaneously coordinate anions to inhibit deprotonation and compete with cations to impede the nucleation process, as shown in Table 1 [76]. By introducing double additives, the operating temperature range of vanadium electrolytes was expanded by 180%, and the energy density was raised by more than 30%, compared to the conventional electrolyte. Apart from optimizing the chemistry of VRFB, an alternative to improve its performance is to enhance its thermodynamics. Taking advantage of the temperature dependence of the cell voltage, Reynard et al. [61] directly converted heat into electrical energy by using the battery as a thermal storage intermediate to achieve a thermally regenerative electrochemical cycle, as shown in Figure 11. The VRFB absorbed heat during charge and emitted it during discharge. In the sulfate–chloride mixed acidic electrolytes, this technique can extend the precipitation time by 8 h at 60 °C without HCl vapor.

#### 4.1.2. Electrostatic Repulsion Agents

Electrostatic repulsion agents rarely exist in inorganic additives. One exception is γ-Al_2_O_3,_ which shows a better anti-precipitation effect at 40 °C and 60 °C, and with which the V(V) concentration retention rate can be improved by nearly 30% [52]. This is mainly attributed to the formation of agglomerated ionic structures after the colloid is generated by the hydrolysis of γ-Al_2_O_3_ combined with V(V) ions. It will enhance the outer layer charge and increase repulsion, thereby achieving high-temperature stability. There are many other additives with stabilizing mechanisms of electrostatic repulsions, such as TiO_2_ and TiOSO_4_, as shown in Table 1 [70].

#### 4.1.3. Growth Inhibitors

Growth inhibitors in inorganic additives mainly include H_2_PO_4_^−^, HPO_4_^2−^, Al_2_O_3_, and sodium hexametaphosphate. Adding NH_4_H_2_PO_4_ and (NH_4_)_2_HPO_4_ can reduce the V_2_O_5_ particle size and narrow its distribution [54]. Specifically, the size of V_2_O_5_ particles was reduced from about 31.7 (±26.6) μm to 18.9 (±13.7) μm and 11.5 (±11.8) μm via NH_4_H_2_PO_4_ and (NH_4_)_2_HPO_4_, respectively. The hydrolysis particles produced by the dissolution of Al_2_O_3_ in the sulfuric acid electrolyte were adsorbed on the surface of the precipitation nuclei, which could effectively inhibit the growth of precipitation [52]. In addition, sodium hexametaphosphate was highly effective in inhibiting the growth rate of crystals by blocking active sites of V_2_O_5_ particles, with a very small concentration (2 ppm) [43].

### 4.2. EMTEs

A proliferation of studies has shown that the introduction of additives plays an important role in boosting the electrochemical reaction kinetics of electrolytes. For example, the addition of H_3_PO_4_ to the negative electrolyte can significantly reduce polarization resistance, improve cell efficiency, and achieve higher electrochemical performance [74]. With the introduction of hydrochloric acid, the average Coulombic efficiency, voltage, and energy efficiency of the VRFB were 3% to 10% higher than those of commercial electrolytes [61]. After adding NaCl as an additive, the energy efficiency, vanadium utilization ratio, and capacity retention ratio, along with Coulombic efficiency, increased by 2%, 4.1%, 12%, and 1%, respectively [64]. This is because additives are capable of compressing the thickness of the double electric layer and reducing the interfacial mass transfer resistance. In addition, employing seawater instead of deionized water as an electrolyte can increase the Coulombic efficiency by 1.2% and reduce the capacity decay rate [68]. The efficiency of the VRFB can also be raised by 2–3% by introducing a very small amount of W^6+^ (3 mM) into the negative electrolyte [65]. The addition of γ-Al_2_O_3_ to the positive electrolyte can make the discharge capacity up to 87.2 mAh, with remarkable electrochemical enhancement, as shown in Table 1 [52]. Introducing sodium molybdate can increase the discharge capacity and energy of the VRFB by 25.59% and 21.89%, respectively [66]. Furthermore, the insertion of TiB_2_ into the primary electrode can enrich defect sites, and the energy efficiency can be increased by 14.06% for 300 cycles [67]. When V(II) ions were adsorbed on the surface of TiB_2_, B acted as an electron acceptor because of its stronger electronegativity. Subsequently, the electron transfer from V(II) to TiB_2_ was accelerated, thus hastening the transition from V(II) to V(III) and improving the reaction kinetics. In addition, some researchers have found excellent enhancement of the reaction kinetics for VA group elements in a deep eutectic solvent electrolyte of the VRFB [77]. Therefore, the application of VA group elements in vanadium electrolytes merits further investigation.

## 5. Organic Additives

Organic additives mainly include weak organic acids, organic salts, and alcohol. They can effectively improve the stability and electrochemical performance of vanadium electrolytes. The organic additives employed in the vanadium electrolytes of different valences in recent years are summarized in Table 2, Table 3, Table 4 and Table 5. As shown in Table 2, Table 3, Table 4 and Table 5, the organics containing one or more functional groups, such as -OH, =O, -COOH, -NH_2_, -SH, and -SO_3_H, contribute to improving the stability and electrochemical mass transfer of the vanadium electrolyte.

### 5.1. Stabilizing Agents

#### 5.1.1. Complexing Agents

It has been demonstrated, as shown in Table 2, Table 3, Table 4 and Table 5, that organic additives such as pyridinium propyl sulfobetaine (PPS), ammonium acetate, 1-hydroxyethane-1,1-diphosphonic acid (HEDP), acidic amino acids, and organophosphorus compounds, can effectively lengthen the precipitation time and widen the temperature range of the vanadium electrolyte [31,44,53,59,79,89,90]. For example, PPS was able to delay the induction time for precipitation by an average of 3–6 h at 50 °C [59]. The addition of 1 wt% HEDP could extend the temperature range of the electrolyte from 0–25 °C to 0–40 °C without influencing the electrochemical activity and cell efficiency. Specifically, HEDP binds with V(V) ions in a molar ratio of 1:1 to form complexes, hindering the first step of the precipitation reaction and suppressing the formation of [VO_2_(H_2_O)_3_]^+^ [79]. The vanadium concentration retention rate of an electrolyte could be successfully raised by 30.53% over the blank after standing for 150 h by adding 3% L-aspartic acid. The -COOH groups carried by L-aspartic acid are easily coordinated with V(IV) and V(V) ions [31]. Furthermore, 3-Aminopropylphosphonic acid (3-APPA) was also found to prolong the precipitation induction time of the electrolyte to 6 days at 45 °C, which is because the -OH, =O, and -NH_2_ groups are capable of encapsulating vanadate ions to form stable penta-coordinated hydrates [44].

#### 5.1.2. Electrostatic Repulsion Agents

Some organic additives, such as compounds with -COOH, -OH, -SO_3_H, -S-, and -NH_2_ functional groups, were found to act as electrostatic repulsion agents in vanadium electrolytes to improve stability. As illustrated in Table 2, Table 3, Table 4 and Table 5, organic compounds such as PAA, L-cystine (LC), methanesulfonic acid (MSA), aminomethanesulfonic acid (AMSA), acidic amino acids, potassium salts of organic carboxylic acids, and carbohydrates could effectively retard precipitation and widen the operating temperature range. For example, adding PAA to vanadium electrolytes prolonged the precipitation time by 6 h. Because the -COOH anion functional groups involved in PAA tend to disassociate with the negatively charged groups: -COO^−^, they generate electrostatic repulsion and intensify dispersion [33]. LC exhibited excellent high/low-temperature stability in negative electrolytes. Specifically, the induction time for the precipitation of vanadium electrolytes can increase by nearly 30 h at 5 °C, and no precipitates were observed in electrolytes for up to 15 days at 50 °C. This achievement of long-term stability can be attributed to strong repulsion arising from -NH_2_ groups in LC, which adsorbs a large amount of H^+^ and creates an external charge layer of -NH_3_^+^, allowing the complexes V(LC)^3+^ to have a higher electrostatic repulsion to each other [53]. The carbohydrate with the highest low-temperature stability was α-lactose monohydrate, and the induction time for precipitation can be extended by 15 h at −20 °C. This is because the hydroxyl and carbonyl groups carried by α-lactose monohydrate attach to the surface of the core of V(II) ions, generating electrostatic repulsion. As a disaccharide with a larger molecular structure, it has a significant steric hindrance, which could effectively enhance the dispersion effect and delay the polymerization of V(II) ions [32].

#### 5.1.3. Growth Inhibitors

There has been very little research on precipitation inhibitors in organic additives. Among the limited research, Kim et al. [58] confirmed that MSA could effectively stabilize the vanadium electrolytes in various valences by inhibiting the growth of V_2_O_5_ precipitation, and the amount of precipitation in the electrolyte was lessened by 50% at 40 °C. However, this effect was not achieved by the role of growth inhibitor, which mainly acted as an electrostatic repulsion agent.

### 5.2. EMTEs

In recent years, a large number of studies have shown that some additives, such as carbohydrates, organic weak acids, and anthraquinones, can be utilized to improve energy efficiency, capacity retention rate, and other electrochemical properties [68,80]. The most common explanation for this is that they contain certain functional groups such as -OH, -COOH, -NH_2_, and -SO_3_H, [31,33,44,53,58,82,85], as shown in Table 2, Table 3, Table 4 and Table 5. For instance, among different kinds of carbohydrates, α-lactose monohydrate performed with the highest capacity retention rate in the charge/discharge tests at low temperatures, with a 41.5% increment over the pristine. Herein, the -OH carried by α-lactose monohydrate provided more active sites for the V(II)/V(III) redox reaction, strengthening the hydrophilicity of the electrode [32]. Furthermore, 3% PAA was capable of raising the capacity retention rate by 23.76% at 50 °C after 100 cycles [44]. The addition of PPS can also increase the capacity retention rate of electrolytes by 5%, and the voltage efficiency along with energy efficiency increased by about 2%. Meanwhile, the electrolyte containing 1% PPS showed the highest reactivity and reversibility [59]. Furthermore, anthraquinone-2,6-disulfonic acid can raise the capacity efficiency of the VRFB by 7.6% [80]. 3APPA and 3PPA-Na promoted redox reactions through the adsorption of oxygen-containing functional groups (e.g., C=O-OH, -NH_2_) on the surface of the graphite felt electrode, and the discharge capacity retention values were improved by 16.3% and 8.3%, respectively [44]. Some additives such as taurine [56], MSA [58], and benzoyl peroxide [60] can be used to modify electrodes to improve their hydrophilicity and simultaneously reduce the zeta potential in the electric double layer. As a result, these additives have demonstrated improved capacity retention, increased average efficiency, increased energy density, and so on.

In contrast to inorganic additives, most organic additives have a higher viscosity, which will cause a lower conductivity of the solution and weaker electrochemical mass transfer, especially for long-chain organics. Therefore, viscosity is also one of the key indicators for designing and selecting additives. Controlling viscosity parameters is of great importance for the improvement of solution fluidity and the enhancement of electrochemical mass transfer [20,53]. In recent years, researchers from the Institute of Metal Research, Chinese Academy of Sciences, have focused on modeling the viscosity of electrolytes with different additives [91,92,93]. Li et al. [92] first proposed a semi-empirical method to estimate the viscosities of ternary mixtures of VOSO_4_·H_2_SO_4_·H_2_O for an electrolyte without extra additives. Compared to their earlier work, [93], the accuracy was greatly improved, and the results aligned well with the experiments involving the average absolute relative deviation (AARD) of 0.96%. Later, they further proposed the ternary solution viscosity model (atmospheric pressure, 283.15–318.15 K) for methanesulfonic acid as an electrolyte additive of VRFBs with the AARD of 0.22% [91]. In this model, the viscosity coefficient, activation energy, and Gibbs free energy of activation were integrated for better accuracy and adaptability. Guo et al. [94] then developed semi-empirical equations for the viscosity of VOSO_4_·PAA·H_2_O ternary solutions at different temperatures, from 283.15 K to 318.15 K, and ionic strengths, and the equations were in good agreement with the experiment results. Moreover, the viscosity of this system descends with the rising temperature and significantly ascends with the rising concentration of PAA.

## 6. Complex Additives

As discussed, the introduction of inorganic and organic additives in the electrolyte is an effective way to improve the stability and electrochemical properties of VRFBs. At the same time, both inorganic and organic additives have their own limitations. Therefore, researchers have tried to develop complex additives composed of inorganic–inorganic additives or inorganic–organic additives to achieve better performance.

For instance, Skyllas-Kazacos [40] prepared a sort of electrolyte containing a mixture of (NH_4_) SO_4_ (2 wt%) and H_3_PO_4_ (1 wt%), which showed the best stability in 22 days at 50 °C. It was also observed that capacity decreased slightly, and energy efficiency remained constant. Li et al. [95] compared three compound additives in positive electrolytes with 1% KHSO_4_ + 3 mmol/L SDBS (Sodium dodecyl benzenesulfonate), 1% KHSO_4_ + 2 mmol/L D-Sorbitol, and 1% KHSO_4_ + 2 mmol/L CTAB (Hexadecyl trimethyl ammonium Bromide) and demonstrated that the combination of 1% KHSO_4_ and 2 mmol/L CTAB was regarded as the most appropriate recipe, instead of adding 1% KHSO_4_ only, extending precipitation time by 5 days and significantly improving electrochemical kinetics. Ren et al. [41] added KHSO_4_ (1%) and SDBS (3 mmol/L) to both positive and negative electrolytes. They found that the concentration of V(V) in the electrolyte was 0.22 mol/L higher than that of the pristine at 45 °C, showing better thermal stability with unchanged electrochemical properties. Moreover, these studies indicated that KHSO_4_ is considered to be an extremely key ingredient, and it is widely applied in compound additives. Ding et al. [19] further verified that the performance of electrolytes will be unaffected by K^+^ with a concentration less than 8.0 × 10^−3^ mol·L^−1^. Li et al. [96] also reported that the inorganic–organic combination of Na_2_SO_4_ and CH_3_CH_2_OH can increase conductivity and improve the stability of the vanadium electrolyte. Further, it was verified that the combination of amino trimethylene phosphonic acid (ATMPA) and hexamethylene diamine tetramethylene phosphonic acid (HDTMPA) can decrease charge transfer resistance of electrolyte by 2.05 Ω cm^2^ and extend precipitation time by 10 days. The mixture of ATMPA and HEDP also performed great effects on stability and electrochemical performance simultaneously, showing a charge transfer resistance reduction of 3.29 Ω cm^2^ and extended precipitation time of 25 days [97].

With few studies on complex additives and many issues remaining to be solved, the future of the practical application of complex additives is still unclear. For one thing, there is still much space for improvement of the reasonable combination ratio of additives; for another, it is unclear whether compound additives can achieve the desired cooperative reinforcement effect because the specific mechanism of multiple additives mixtures may differ from that of independent additives.

## 7. Side Effects of Additives

Some inorganic additives have side effects, such as acid blends that allow the electrolyte to have a higher vanadium concentration and a wider operating temperature range. However, high concentrations of mixed acids can drive up the cost of the VRFB or may increase the risk of material corrosion. The chlorine-containing additives can also generate Cl_2_ at high potentials, which can cause unfavorable precipitation between the anions and the low-valence vanadium ions, resulting in contamination of the operating environment and reduced energy density of the VRFB.

Some organic additives mainly face the problem of poor life-span, and organic additives containing -OH/-COOH/=O groups have been proven to be unsuitable for use in cathode electrolytes due to their low long-term stability in cathode electrolytes with strong acidic and oxidizing properties during the long-term cycling of VRFB. In addition, organic compounds containing -SO_3_H/-NH_2_ hydrophilic groups, such as taurine and aminomethane sulfonic acid, which are commonly used as cathode additives, still have a handful of limitations. For instance, the synthesis of long-chain polymers through intermolecular hydrogen bonding (-SO_3_—^+^H_3_N-) in an acidic electrolyte and protonation (-NH_3_^+^) may lower the concentration of H^+^ ion in the redox reaction. Such drawbacks result in a lower efficiency of additives and reduced positive electrolyte stability.

One more prominent point is that many studies have shown that the excess of additives is detrimental to the stability improvement of vanadium electrolytes. At the molecular level, adsorption competition between vanadium ions and additives would be formed due to the limited binding sites on the surface of the electrode plate, leading to a drop in redox current and capacity [68,73]. From the perspective of ions or functional groups, excessive additives would cause the alteration of the mechanism of interaction between V(V) and ions or functional groups including -OH, -COOH, Cl^−^, and so on. For instance, the hydrated layer of vanadium ions would be disrupted, forming different kinds of neutral molecules, which can promote the nucleation of additional precipitates [33,34]. Furthermore, there is a possibility that the vanadium ions would be coated, making it difficult to participate in the reactions on the electrode surface and disturbing ionic behaviors inside the electrolyte. Furthermore, some reducing functional groups can also bring about the instant reduction of V(V) ions, which can reduce the concentration of vanadium ions involved in the redox reaction of VRFBs, leading to the deterioration of the performance and life cycle of VRFBs [44]. Other hydrophobic groups also can hinder the diffusion of ions in the interface of electrolyte and electrode, worsening the electrochemical performance while improving the stability [59]. As a consequence, the addition is one of the major factors affecting electrochemical performance enhancement.

For example, when the electrolyte composition of a chloride ion concentration is 6.0–7.0 M and the sulfate concentration is 2.0–3.0 M, an electrolyte with a sulfate concentration of 2.75 M and a chloride ion concentration of 5.8 M performed best. In this case, the VRFB could achieve stable operation over a wide temperature range (20–50 °C) [98,99]. Liu et al. [88] noticed that DL-malic acid is less stable than L-aspartic acid in the vanadium electrolyte because the former has three -OH and the latter has two. The excess -OH in DL-malic acid caused the formation of new precipitates, resulting in poor stabilization. As shown in Table 2, phosphonic acid, ethylenediaminetetraacetic acid (EDTA), and N-(phosphonomethyl) iminodiacetic hoursydrate with the excess -OH performed the worst in terms of battery performance. As reported by Wei et al. [59], although ESA is structurally similar to PPS and TA, it did not enhance the electrochemical transfer to the electrolyte because it has alkyl groups, a type of hydrophobic group.

## 8. Summary and Prospects

To conclude, additives are a double-edged sword for VRFBs. On the one hand, as an additional component introduced in an all-vanadium liquid flow battery, whether the additive is recyclable in the system and how it should be recycled deserve critical consideration. If it is necessary to introduce an additive into the vanadium electrolyte, it has to meet the long-term stability requirements and be unable to be degraded in this system over time. Meanwhile, the adoption of additives for positive or negative electrolytes with respect to VRFBs only is less desired, because it destroys the non-cross contamination properties of VRFBs, and the practical application is limited by the penetration of the additives. Furthermore, it is unclear whether there is corrosion or a slow reaction of the additive to electrodes made of carbonaceous materials and the ion-exchange membrane made of organic material with the long-term presence of the additive in the electrolyte. All in all, a series of possible issues brought about by the introduction of additives to the electrolyte system should be taken into account.

On the other hand, additives can expand the temperature window of VRFBs and enhance the electrochemical reaction kinetics, with a low dosage realizing an excellent effect. They are considered to be a key factor in accelerating the industrialization of the VRFB in the future. In recent years, research on the electrolyte additives of VRFBs has made great progress, but there are still problems that need to be solved, such as finding more efficient recipes for compound additives and figuring out the microcosmic mechanisms of different additives. A large number of studies on the additives’ mechanisms have shown that complexation is the core mechanism to enhance the stability of vanadium electrolyte, along with electrostatic repulsion and growth inhibition. Optimization of the electrolyte–electrode interface is the key to improving the electrochemical reaction kinetics of vanadium batteries. Specifically, in order to further enhance the overall performance of vanadium electrolyte by additives, ensuring that the additive functions without adverse effects on both positive and negative electrolytes and maintains non-cross contamination properties, and designing additives from multiple aspects, including electrolyte, electrode, and membrane, to enhance vanadium electrolyte performance should be considered, such as loading an oxygen-containing functional group on the electrode [100,101]. Ultimately, to successfully achieve a broadened operating temperature for VFB systems, it is necessary to find an additive that would not have a significant impact on the corrosion and cost of the electrolyte, and that would simultaneously improve the stability and electrochemical performance of the positive or negative electrolyte without significant adverse effects.

## Figures and Tables

**Table 1:** Common inorganic additives in vanadium electrolytes.

Additives	Temp. (°C)	Electrolyte	Stabilizing Effect	Electrochemical Properties	Categories	Mechanism	Notes	Ref.	Year
HCl	20–60	positive	Precipitation extension time: 18 h (60 °C)	/	Stabilizing agents	Complexation	/	[61]	2018
Fe_2_(SO_4_)_3_	25–50	positive	Precipitation extension time: 168 h (50 °C), Increased V(V) concentration retention rate: 34% (approximately)	/	Stabilizing agents	Unclear	/	[62]	2019
KH_2_AsO_4_	30–70	positive	Precipitation extension time: 146 h (45 °C)	/	Stabilizing agents	Maybe complexation	With toxicity	[63]	2019
Cl^−^	25	positive	/	Improved capacity retention: 12% (approximately), increased energy efficiency: 2%	Electrochemical mass transfer enhancers	Increased active sites	/	[64]	2021
W^6+^	25	negative	/	Improved efficiency: 2–3%	Electrochemical mass transfer enhancers	Increased active sites	/	[65]	2017
Na_2_MoO_4_	−10	negative	/	Increased discharge capacity: 25.59%, discharge energy: 21.89%, increased energy efficiency: 1.61%, increased Coulombic efficiency: 1.97%	Electrochemical mass transfer enhancers	Increased active sites	/	[66]	2018
TiB_2_	25	electrode	/	Increased energy efficiency: 14.06%	Electrochemical mass transfer enhancers	Increased active sites	^/^	[67]	2022
NaCl	25	negative	/	Increased Coulombic efficiency: 1.2%	Electrochemical mass transfer enhancers	Increased active sites	Seawater as an alternative to deionized water mainly for lowering the cost	[68]	2019
NaCl	25–50	positive	Precipitation extension time: 42 h (50 °C)	/	Stabilizing agents + Electrochemical mass transfer enhancers	Complexation + Increased active sites	^/^	[69]	2018
Na_2_HPO_4_	5–50	positive	Precipitation extension time: 180 h (50 °C)	Improved capacity retention: 10% (approximately)	Stabilizing agents + Electrochemical mass transfer enhancers	Complexation + Increased active sites	/	[30]	2015
NH_4_H_2_PO_4_	5–50	positive	Precipitation extension time: 120 h (50 °C)	Increased capacity efficiency: 10% (approximately)	Stabilizing agents + Electrochemical mass transfer enhancers	Complexation + Increased active sites	/	[30]	2015
TiO_2_	25–60	positive	Increased V(V) concentration retention rate: 10% (60 °C)	/	Stabilizing agents + Electrochemical mass transfer enhancers	Electrostatic repulsion + Increased active sites	/	[70]	2017
TiOSO_4_	25–60	positive	Increased V(V) concentration retention rate: 15% (60 °C)	/	Stabilizing agents + Electrochemical mass transfer enhancers	Electrostatic repulsion + Increased active sites	/	[70]	2017
γ-Al_2_O_3_	25–60	positive	Increased V(V) concentration retention rate: 20% (60 °C)	Increased discharge capacity: 13% (approximately)	Stabilizing agents + Electrochemical mass transfer enhancers	Electrostatic repulsion + Increased active sites	The performance of γ-Al_2_O_3_ is better than α-Al_2_O_3_	[52]	2017

**Table 2:** Organic additives for V(V) solution.

Additives	V(V)/M	S/M	Temp. (°C)	Amounts	Functional Groups	Stabilizing Effects	Electrochemical Properties	Categories	Mechanisms	Ref.	Year
L-glutamic acid	2	3	40	1–5 wt%	-OH, -NH_2_	Increased V(V) concentration retention rate: 16.1%	/	Stabilizing agents	Electrostatic repulsion	[57]	2013
Serine	2	3	50	2 wt%	-COOH, -NH_2_	Precipitation extension time: 1 h (50 °C)	/	Stabilizing agents	Complexation	[78]	2014
3-Phosphonopropion-ic acid (3PA)	2	3	45	0.1 M	-OH, =O	Precipitation extension time: 2 days (45 °C)	/	Stabilizing agents	Electrostatic repulsion	[44]	2018
1-hydroxyethane-1,1-diphosphonic acid (HEDP)	2–2.5	2	15~45	1 wt%	-OH, =O	Temperature range is widened from 0–25 °C to 0–40 °C	/	Stabilizing agents	Complexation	[79]	2020
Anthraquinone-2,6-disulfonic acid (AQDS)	1	2	25	Unclear	=O, -SO_3_H	/	Increased capacity efficiency: 7.6%	Electrochemical mass transfer enhancers	Increased active sites	[80]	2017
Taurine	1.5	3	40	0.1 M	-SO_3_H, -NH_2_	Increased V(V) concentration retention rate: 6%	Improved capacity retention: 5%, increased energy density: 5%	Stabilizing agents + Electrochemical mass transfer enhancers	Complexation+ Increased active sites	[56]	2018
Taurine	2	3	40	4 mol%	-SO_3_H, -NH_2_	Precipitation extension time: 54 h (40 °C)	Increased the average energy efficiency: 3%	Stabilizing agents + Electrochemical mass transfer enhancers	Complexation+ Increased active sites	[81]	2018
Methanesulfonic acid	2	2	−5~40	1/2 M	-OH	Precipitation extension time: 7 days (45 °C)	Improved capacity retention: 30%, increased energy efficiency: 3%, increased Coulombic efficiency: 3%	Stabilizing agents + Electrochemical mass transfer enhancers	Electrostatic repulsion/Growth inhibition + Increased active sites	[58]	2021
Sodium 3-phosphonopropion-ate (3PPA-Na)	2	3	45	0.1 M	-OH, =O, -COOH	Precipitation extension time: 2 days (45 °C)	Improved capacity retention, improvement: 16%	Stabilizing agents + Electrochemical mass transfer enhancers	Growth inhibition + Increased active sites	[44]	2018
3-Aminopropylphosp-honic acid (3APPA)	2	3	45	0.1 M	NH_2_, -OH	Precipitation extension time: 4 days (45 °C)	Improved capacity retention, improvement: 8%	Stabilizing agents + Electrochemical mass transfer enhancers	Complexation + Increased active sites	[44]	2018
Potassium daysiformate	2.876	6	−5~60	0.5 wt%	-COOH	Precipitation extension time: 10 h (45 °C), increased V(V) concentration retention rate: 11%	Increased energy efficiency: 2%	Stabilizing agents + Electrochemical mass transfer enhancers	Electrostatic repulsion + Increased active sites	[82]	2022
Aspartic acid	2	3	50	3 wt%	-COOH, -NH_2_	Increased V(V) concentration retention rate: 30.53%	Increased energy efficiency: 4%	Stabilizing agents + Electrochemical mass transfer enhancers	Complexation + Increased active sites	[31]	2019
LC	1.8	3	50	2 wt%	-OH, -NH_2_, =O	No found precipitate	Improved capacity retention: 1%, increased energy efficiency: 5%	Stabilizing agents + Electrochemical mass transfer enhancers	Complexation + Increased active sites	[53]	2019
Phosphonic acid, Ethylenediaminetet-raacetic acid (EDTA), N-(phosphonomethyl) iminodiacetic hoursydrate, etc.	2	3	45	0.1 M	-OH, =O	Instant reduction	/	Counterproductive	The excess of -OH made V(V) instantly reduced by additives	[44]	2018

**Table 3:** Organic additives for V(IV) solution.

Additives	V(IV)/M	S/M	Temp. (°C)	Amounts	Functional Groups	Stabilizing Effects	Electrochemical Properties	Categories	Mechanisms	Ref.	Year
LC	1.8	3	5	2%	OH, =O, -NH_2_	Precipitation extension time: 104 h (5 °C)	/	Stabilizing agents	Complexation	[15]	2019
Quinolinic acid	2	3	40	1%	-COOH, pyridyl	/	Improved capacity retention: 2.18%, increased the average energy efficiency: 1.04%	Electrochemical mass transfer enhancers	Increased active sites	[83]	2014
Histidine	1.5	3	40/60	0.05 M	-COOH, -NH_2_	Increased V(V) concentration retention rate: 18% (40 °C)	Improved capacity retention: 45.5%, increased energy efficiency: 7.5%	Stabilizing agents + Electrochemical mass transfer enhancers	Increased active sites	[84]	2019
Aspartic acid	2	3	50	3~4%	-COOH, -NH_2_	Increased V(V) concentration retention rate: 30.53%	Increased energy efficiency: 4%	Stabilizing agents + Electrochemical mass transfer enhancers	Electrostatic repulsion + Increased active sites	[31]	2019
PAA	1.89	3	50	3%	-COOH	Precipitation extension time: 4 h (50 °C)	Improved capacity retention: 23.76%	Stabilizing agents + Electrochemical mass transfer enhancers	Electrostatic repulsion + Increased active sites	[33]	2019
AMSA	2	3	50	1%	-NH_2_, -SO_3_H	Increased V(V) concentration retention rate: 20% (approximatel)	Improved capacity retention: 7.6%	Stabilizing agents + Electrochemical mass transfer enhancers	Electrostatic repulsion + Increased active sites	[85]	2013
Taurine	1.98	3	40	1%	-NH_2_, -SO_3_H	Precipitation extension time: 24 h (40 °C)	Improved energy density: 28.9 Wh·L^–1^	Stabilizing agents + Electrochemical mass transfer enhancers	Electrostatic repulsion	[86]	2023
Acetic acid	2	3	−25	1%	-COOH	Precipitation extension time: 95 h (−25 °C)	Improved energy density: 25.5 Wh·L^–1^	Stabilizing agents + Electrochemical mass transfer enhancers	Electrostatic repulsion	[86]	2023
Ammonium acetate	1.9	3	25	0.5%	-COOH, -NH_2_	No found precipitate in 30 days	The impedance of the electrode reaction is reduced	Stabilizing agents + Electrochemical mass transfer enhancers	Complexation	[87]	2016

**Table 4:** Organic additives for V(III) solution.

Additives	V(III)/M	S/M	Temp. (°C)	Amounts	Functional Groups	Stabilizing Effects	Electrochemical Properties	Categories	Mechanisms	Ref.	Year
DL-malic acid	2	3	4/25	Unclear	-OH, =O, -NH_2_	Precipitation extension time: 22 h (25 °C), 17 h (4 °C)	/	Stabilizing agents	Complexation	[88]	2014
LC	1.8	3	5/50	2 wt%	-OH, =O, -NH_2_	Precipitation extension time: 6~62 h (5 °C)	/	Stabilizing agents	Complexation	[53]	2019
Glucose	1.8	3	−20/25	1 wt%	-OH	Maintaining stability: 30 days (−20 °C)	/	Stabilizing agents	Grow inhibition	[32]	2022
Sucrose	1.8	3	−20/25	1 wt%	-OH	Maintaining stability: 30 days (−20 °C)	/	Stabilizing agents	Complexation	[32]	2022
D(+)-xylose	1.8	3	−20/25	1 wt%	-OH	Maintaining stability: 30 days (−20 °C)	/	Stabilizing agents	Grow inhibition	[32]	2022
L-aspartic acid	2	3	4/25	Unclear	-OH, =O, -NH_2_	Precipitation extension time: 168 h (25 °C); 114 h (4 °C)	Increased Coulombic efficiency: 1.2%, increased energy efficiency: 2.6%	Stabilizing agents + Electrochemical mass transfer enhancers	Electrostatic repulsion+ Increased active sites	[88]	2014
α-lactose monohydrate	1.8	3	−20/25	1 wt%	-OH	Maintaining stability: 30 days (−20 °C)	Improved capacity retention: 41.5% (30 cycles)	Stabilizing agents + Electrochemical mass transfer enhancers	Complexation + Increased active sites	[32]	2022

**Table 5:** Organic additives for V(II) solution.

Additives	V(II)/M	S/M	Temp. (°C)	Amounts	Functional Groups	Stabilizing Effects	Categories	Mechanisms	Notes	Ref.	Year
MSA	2	2	−5	0.5 M	-SO_3_H	Precipitation extension time: 30 days (−5 °C)	Stabilizing agents	Growth inhibition	/	[58]	2021
Glucose	1.8	3	−20/25	1 wt%	-OH	Precipitation extension time: 3 h (−20 °C)	Stabilizing agents	Electrostatic repulsion	/	[32]	2022
Sucrose	1.8	3	−20/25	1 wt%	-OH	Precipitation extension time: 7.5 h (−0 °C)	Stabilizing agents	Electrostatic repulsion	/	[32]	2022
D(+)-xylose	1.8	3	−20/25	1 wt%	-OH	Precipitation extension time: 2 h (−20 °C)	Stabilizing agents	Electrostatic repulsion	/	[32]	2022
α-lactose monohydrate	1.8	3	−20/25	1 wt%	-OH	Precipitation extension time: 15 h (−20 °C)	Stabilizing agents	Electrostatic repulsion	Optimal low-temperature stability in carbohydrate	[32]	2022
LC	1.8	3	5	2 wt%	-OH, -NH_2_, =O	Precipitation extension time: 60 h (5 °C)	Stabilizing agents	Complexation	/	[53]	2019

## Data Availability

Not applicable.
